# Long-term follow-up of motor function deterioration following microsurgical resection of middle third parasagittal and falx meningioma

**DOI:** 10.1186/s41983-018-0013-3

**Published:** 2018-04-25

**Authors:** Ashraf Ahmad Elzarief, Mohamed Fouad Ibrahim

**Affiliations:** Neurosurgery department, Cairo University, Cairo, Egypt

**Keywords:** Surgical results, Parasagittal meningioma, Falx meningioma

## Abstract

**Background:**

Incidence of parasagittal meningioma varies in literature; it ranges from 16.8% to 25.6% of intracranial meningioma. Parafalcine meningioma accounts for about 8.5% of intracranial meningioma. Based on their relation to the superior Sagittal sinus and falx, these tumors had been classified into 3 groups; anterior third located between crista galli and coronal suture, middle third located between coronal and lambdoid sutures, and posterior third located posterior to lambdoid suture.

**Methods:**

Seventeen cases of middle third parasagittal and falx meningioma operated between 2010 and 2014 were retrospectively reviewed; extent of resection was expressed according to Simpson’s classification. Medical Research Council Grading System was used for assessment and evaluation of *motor power* during preoperative, postoperative, and long-term follow-up, and patients were divided into two groups: group A, no preoperative motor deficit, and group B, patients with preoperative motor deficit. Based on this grading system, we classified *motor function* into three categories as follows: *no disability*, *partial disability but independent*, and *complete disability*. Follow-up period ranged between 14 and 48 months with mean period 32 months.

**Results:**

Total number of patients was 17, 10 females and 7 males. Age ranged between 38 and 63 with the mean age 47. Twelve cases were parasagittal meningioma and 5 cases were falx meningioma. All located at the middle one third. Family history was negative in all cases. Duration of presenting symptoms varied between 3 and 28 months; presenting symptoms were as follows: seizures 64.7% (11 patients), headache 52.9% (9 patients), motor weakness 47% (8 cases), and disturbed conscious level 5.9% (1 case). According to Simpson’s classification, grade I resection was obtained in 4 patients and grade II in 13 patients. Intraoperative sinus invasion was present in 3 patients. In early postoperative outcome regarding motor function, 9 cases (53%) showed deterioration of motor function in group A: 4 patients out of 9, and group B: 4 patients out of 8. On long-term follow-up of patients with deteriorated motor function, 6 patients out of 9 improved (66%).

**Conclusions:**

Parasagittal and falx meningioma involving the middle third is associated with a higher incidence of motor function deterioration either as a presenting symptom or during postoperative period. Adobting the microsurgical techniques during surgical resection and preservation of integrity of the venous system and cerebral cortex, deterioration of motor function is transient in most of cases with a favorable outcome on long-term follow-up.

## Background

Incidence of parasagittal meningioma varies in literature; it ranges from 16.8 to 25.6% of intracranial meningioma. Parafalcine meningioma accounts for about 8.5% of intracranial meningioma (Sang-Bong et al., 2007; Wilkins, 1991). Based on their relation to the superior sagittal sinus and falx, these tumors had been classified into three groups: anterior third located between crista galli and coronal suture, middle third located between coronal and lambdoid sutures, and posterior third located posterior to lambdoid suture (Hossly & Olivecrona, 1955). Being benign lesions with a supratentorial location, it seems that total surgical resection is curative; however, it carries a high risk of venous infarction (Sindou & Hallacq, 1996; Sindou, 2001), and early outcome of surgical resection of these lesions in most of series seems unsatisfactory with a high rate of postoperative complications. Poor postoperative outcome regarding motor power and function in cases of surgically excised middle third parasagittal and parafalcine meningiomas in which transient or permanent hemiparesis developed, is one of the most frequent complications (Skudas & Tamasauskas, 2002). This study is focused on evaluation of motor function deterioration following microsurgical resection of middle third parasagittal and falx meningioma, and results of long-term follow-up.

## Methods

A number of 17 cases of middle third parasagittal and falcine meningiomas operated between 2010 and 2014 were retrospectively reviewed; all tumors were attached to the middle one third of falx or superior sagittal sinus (SSS); all cases proven pathologically to be meningioma.

All data of the cases were reviewed including age and sex. The main aim was evaluation of postoperative motor function changes on short- and long-term follow-up. Recurrent cases or cases which showed recurrence were not included in this study.

### Surgical technique

All 17 cases were operated upon at Cairo University Hospitals by the authors. Supine position was used in cases of unilateral lesions while semi-setting position was used in cases with bilateral lesion (dumbbell-shaped) with different modification according to tumor size, aiming to bring the tumor at the top of the operative field with adequate visualization and exposure.

In unilateral cases, a U-shaped skin flap was based inferiorly, made wide enough to allow adequate exposure, while a bicoronal incision was made in cases with bilateral extension. Bone flap was designed to allow adequate tumor exposure; dural opening was performed to be beyond the edges of the lesion and based on SSS. After dural opening, careful dissection of dura from the underlying cortex was done with a special attention to preserve all draining veins. Internal debulking of the lesion was started followed by dissection of the external capsule of the lesion from the surrounding brain tissue. Tumor parts attached to falx or SSS were the last portion to be removed, in cases where these parts were adherent to the falx cerebri or SSS without invading its cavity coagulation with bipolar was done. In cases with sinus invasion, resection of the tumor within the cavity and sinus wall was done; cases with dural invasion were subjected to duroplasty using fascia lata graft.

**Fig. 1:**
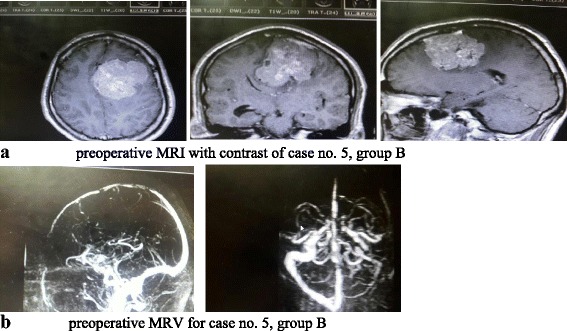
**a** Preoperative MRI with contrast of case no. 5, group B. **b** Preoperative MRV of case no. 5, group B

**Fig. 2:**
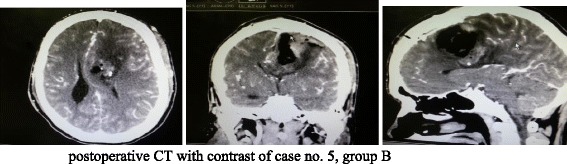
Postoperative CT with contrast of case no. 5, group B

Next day of surgery, radiological and neurological assessment was done. CT brain with contrast was the standard radiological study used to evaluate extent of resection in early postop. period (Figs. 1 and 2).

The Simpson Grading System was used to describe the extent of resection (Simpson, 1957) Table 1.

**Table 1:** Simpson Grading System

Grade	Definition of corresponding resection
I	Macroscopically complete resection with excision of dural attachment and abnormal bone
II	Macroscopically complete resection with coagulation of dural attachment
III	Macroscopically complete resection without resection or coagulation of its attachment
IV	Subtotal resection
V	Simple decompression of the tumor

For evaluation and follow-up of motor function, we classified patients into two groups:

*group A*, patients with no preoperative motor deficit, and *group B*, patients with preoperative motor deficit.

The Medical Research Council Grading System was used for assessment and evaluation of motor power during preoperative, postoperative, and long-term follow-up. Based on this grading system, we classified *motor function* into three categories as follows:

A) *No disability*: motor power is more than grade IV for lower limbs and grade III for upper limbs
B) *Partial disability but independent*: motor power is at least grade IV for lower limbs and grade III for upper limbs
C) *Complete disability*: motor power is less than grade IV for lower limbs and grade III for upper limbs

Follow-up period ranged between 14 and 48 months with a mean period of 32 months.

## Results

The total number of patients was 17, 10 females and 7 males. Age ranged between 38 and 63 with the mean age 47. Twelve cases were parasagittal meningiomas and 5 cases were falx meningiomas. All were located at the middle one third. Family history was negative in all cases. Duration of presenting symptoms varied between 3 and 28 months; presenting symptoms were seizures 64.7% (11 patients), headache 52.9% (9 patients), motor weakness 47% (8 cases), and disturbed conscious level 5.9% (1 case) (Table 2).

**Table 2:** Incidence of presenting symptoms

Presenting symptom	Number of patients	Percentage
Seizures	11	64.7
Headache	9	52.9
Motor weakness	8	47
Disturbed conscious level	1	5.9

According to Simpson’s classification, grade I resection was obtained in four patients while grade II was obtained in 13 patients. Intraoperative sinus invasion was present in 3 patients; in these 3 cases, the tumors within the sinus cavity and the invaded sinus wall were excised and defect in sinus was closed. In 2 cases, we used direct sutures to close the gap, and gel foam was applied, while in the third case, an artificial dural graft was used to close the sinus defect. Histological types of the tumors were transitional in 7 patients, fibroblastic in 6 patients, and meningiothelial in 4 patients (Table 3).

**Table 3:** Results of histopathological examination of excised lesions

Histopathology	Number of patients	Percentage
Transitional	7	41.2
Fibroblastic	6	35.3
Meningiothelial	4	23.5

In early postoperative outcome regarding motor function, 9 cases (53%) showed deterioration of motor function as follows: group A: among 9 patients with no preoperative motor deficit, 4 patients had no deterioration of motor power while 5 patients deteriorated; 3 cases had partial deficit and independent, while 2 cases had complete disability.

In group B: 8 patients with preoperative motor deficit were classified as follows: 5 cases had partial deficit but independent and 3 cases had complete disability, 1 of 5 patients with partial deficit but independent showed improvement of motor power, 4 cases deteriorated and got complete disability, and the remaining 3 cases with preoperative complete disability showed no change of motor power.

Postoperative radiological studies of the deteriorated patients revealed severe cerebral edema in 3 patients and hemorrhagic infarction in 1 patient, while in the remaining 5 cases there were no postoperative radiological changes that could be attributed to their deficits.

On long-term follow-up of patients with deteriorated motor function, 6 patients out of 9 improved (66%):

Group A: 2 of 3 patients with partial deficit but independent showed improvement of motor power and became intact, 1 patient did not improve, and 1 of 2 patients with complete disability improved to partial deficit but independent, while the other case was still the same.

Group B: 3 of 4 cases with complete disability showed improvement of motor power to be partial deficit but independent, while the fourth case did not improve (Tables 4 and 5).

**Table 4:** Motor function evaluation among group A

Case	Preoperative	Early postoperative	Long-term follow-up
1	N	P	N
2	N	N	N
3	N	C	C
4	N	N	N
5	N	P	P
6	N	P	N
7	N	N	N
8	N	N	N
9	N	C	P

Abbreviations: *N* no disability, *P* partial disability but independent, *C* complete disability

**Table 5:** Motor function evaluation among group B

Case	Preoperative	Early postoperative	Long-term follow-up
1	C	C	C
2	P	C	P
3	P	N	N
4	C	C	C
5	P	C	P
6	P	C	P
7	P	C	C
8	C	C	C

No postoperative recurrence detected within the follow-up period that ranged between 14 to 48 months with mean period 32 months

Abbreviations: *N* no disability, *P* partial disability but independent, *C* complete disability

## Discussion

Based on their dural attachments, parasagittal meningiomas are considered as lesions attached to the superior sagittal sinus, while parafalcine meningiomas arise from falx and concealed completely by the overlying cortex, and typically, they do not involve superior sagittal sinus. Incidence of parasagittal meningiomas varies in literature from 16.8 to 25.6 of intracranial meningioma (Wilkins, 1991). The incidence of parafalcine meningioma is less frequent than parasagittal meningiomas; some authors considered falcine meningiomas are five to seven times less common than parasagittal meningiomas (Claus et al., 2005). Akira et al. (2012) reported 16 cases in their study, 12 cases were parasagittal while 4 cases were parafalcine meningioma. Shiro et al. (1990) reported the following incidence of parafalcine and parasagittal meningiomas at the middle and posterior one third: among 15 cases involving the middle third, 3 cases were parafalcine while 12 were parasagittal; in the posterior one third, one case was a parafalcine meningioma while 5 cases were parasagittal. In our study among 17 cases, 12 were parasagittal and 5 were parafalcine.

The main goal of surgical management of parasagittal and parafalcine meningiomas involving the middle third is to do complete excision and protect structures related to motor function, mainly the central gyrus, Rolandic vein, and superficial cortical draining veins; this seems to be not usually visible as in most of cases many difficulties were faced during surgery like large-sized tumors with high vascularity, sinus invasion, and involvements of major cortical veins. In our study, we achieved total resection of the lesion in all cases, according to Simpson’s classification, grade I resection was obtained in 4 patients and grade II was obtained in 13 patients. Intraoperative sinus invasion was present in 3 patients. We did not face recurrence within the follow-up period; this could be due to total resection obtained in all cases and no atypical changes or malignancy found in histopathological examination of excised lesions.

We focused in this study on motor power and function deterioration during early postoperative period and long-term follow-up as parasagittal and parafalcine meningiomas involving middle third are usually associated with a higher incidence of motor power deterioration, either as a presenting symptom or a postoperative complication. In a study done by Jian et al. (2013), the incidence of motor weakness as a presenting symptom was 61%. Shiro et al. (1990) reported an incidence of 40% in their study, while incidence was 0% among lesions involving anterior and posterior third in the same study. In our study, motor power deterioration was the presenting symptoms in 8 patients (47%), 5 cases classified as partial disability but independent (P), and 3 cases had complete disability (C).

Regarding motor function, many authors documented poor results during early postoperative period. Akira et al. (2012) reported an incidence around 50% in their study, where 8 patients out of 16 developed deterioration of motor power during early postoperative period, 6 cases showed hemiparesis, 5 of them had complete hemiplegia, and 2 cases showed monoparesis of the lower limb. Jian et al. (2013) reported that 56% of their patients with preoperative motor deficits developed worsening of motor function during early postoperative period (9 patients out of 16). In our study, in spite of doing all attempts to preserve and protect draining veins, peritumoral brain tissues, and superior sagittal sinus patency and integrity (starting from doing a wide craniotomy flap till following microsurgical technique for tumor resection), early postoperative outcome regarding motor power and function was around 53% (9 cases deteriorated out of 17). Among group A (9 patients with no deficit), 5 patients developed new motor deficits, and among group B (8 patients with previous motor deficit), 4 patients got more worsening of their deficits.

Venous system injury with subsequent cerebral edema, venous infarction, cortical injuries, and contusions are considered the main reasons for poor postoperative outcome regarding motor function (Tomasello et al., 2013; Bazzao et al., 2005; Elborady & Kamal, 2014). However, in many cases, there is no pathology detected in postoperative radiological studies (Na et al., 2013). Akira et al. (2012) reported that in their series in spite of 8 patients out of 16 who developed deterioration of motor power during early postoperative period, only radiological studies were positive in 2 cases only (one case showed intracerebral hematoma and the other showed severe cerebral edema). In our study, postoperative radiological studies were negative in 5 cases with motor power deterioration, while 3 cases showed severe cerebral edema that required intensive medical therapy, including deep sedation and ventilation, one case showed postoperative hemorrhagic infarctions subjected to medical treatment and no surgical intervention was required for any deteriorated cases.

On long-term follow-up of patients with deteriorated motor function, 6 patients out of 9 improved (66%) as follows: in group A, 2 cases (case nos. 1 and 6) with partial deficit but independent showed improvement of motor power and showed no disability, and one patient did not improve (case no. 5). One patient (case no. 9) with complete disability improved and had partial deficit but independent, while the other case still the same (case no. 3). in group B: 3 patients (case nos. 2, 5, and 6) of 4 cases with complete disability showed improvement of motor power to be partial deficit but independent, while the fourth case (case no. 7) did not improve.

Among these 3 cases with permanent complete disability, the first case (case no. 5 in group A) was a 56-year-old male patient with right parasagittal meningioma presenting with seizures and headache with no preoperative motor deficit; on the next day of surgery, the patient developed deterioration of conscious level, GCS became 12, rapid deterioration of motor power (upper limb grade II, Lower limb grade I), CT brain revealed severe cerebral edema, and patient was subjected to aggressive medical therapy including barbiturate coma and ventilation. On the third day, consciousness improved; GCS became 14; on discharge, patient was fully conscious, but motor power was still the same; on long-term follow-up despite that motor power showed mild improvement, upper limb became grade III and lower limb became grade II; and patient was still considered having complete disability. In the other 2 patients (case no. 3 in group A and case no. 7 in group B), no postoperative radiological changes were found.

## Conclusions

Parasagittal and falx meningioma involving the middle third is associated with a higher incidence of motor function deterioration either as a presenting symptom or during postoperative period. Adobting the microsurgical techniques during surgical resection and preservation of integrity of venous system and cerebral cortex, deterioration of motor function is transient in most of cases with a favorable outcome on long-term follow-up.

## Abbreviations

C: Complete disability
N: No disability
P: Partial disability but independent
SSS: Superior sagittal sinus
